# Age‐specific severity of severe acute respiratory syndrome coronavirus 2 in February 2020 to June 2021 in the Netherlands

**DOI:** 10.1111/irv.13174

**Published:** 2023-08-22

**Authors:** Pieter T. de Boer, Jan van de Kassteele, Eric R. A. Vos, Liselotte van Asten, Dave A. Dongelmans, Arianne B. van Gageldonk‐Lafeber, Gerco den Hartog, Agnetha Hofhuis, Fiona van der Klis, Dylan W. de Lange, Lenny Stoeldraijer, the RIVM COVID‐19 epidemiology and surveillance team, Hester E. de Melker, Eveline Geubbels, Susan van den Hof, Jacco Wallinga

**Affiliations:** ^1^ Center for Infectious Disease Control National Institute for Public Health and the Environment (RIVM) Bilthoven The Netherlands; ^2^ Department of Intensive Care Medicine Amsterdam UMC (location AMC) Amsterdam The Netherlands; ^3^ Amsterdam Public Health Research Institute Amsterdam The Netherlands; ^4^ Laboratory of Medical Immunology Radboudumc Nijmegen The Netherlands; ^5^ Intensive Care, University Medical Center Utrecht University of Utrecht Utrecht The Netherlands; ^6^ Statistics Netherlands The Hague The Netherlands; ^7^ Department of Biomedical Data Sciences Leiden University Medical Center Leiden The Netherlands

**Keywords:** COVID‐19, disease severity, epidemiology, infection fatality ratio, SARS‐CoV‐2

## Abstract

**Background:**

The severity of Severe Acute Respiratory Syndrome Coronavirus 2 infection varies with age and time. Here, we quantify how age‐specific risks of hospitalization, intensive care unit (ICU) admission, and death upon infection changed from February 2020 to June 2021 in the Netherlands.

**Methods:**

A series of large representative serology surveys allowed us to estimate age‐specific numbers of infections in three epidemic periods (late‐February 2020 to mid‐June 2020, mid‐June 2020 to mid‐February 2021, and mid‐February 2021 to late‐June 2021). We accounted for reinfections and breakthrough infections. Severity measures were obtained by combining infection numbers with age‐specific numbers of hospitalization, ICU admission, and excess all‐cause deaths.

**Results:**

There was an accelerating, almost exponential, increase in severity with age in each period. The rate of increase with age was the highest for death and the lowest for hospitalization. In late‐February 2020 to mid‐June 2020, the overall risk of hospitalization upon infection was 1.5% (95% confidence interval [CI] 1.3–1.8%), the risk of ICU admission was 0.36% (95% CI: 0.31–0.42%), and the risk of death was 1.2% (95% CI: 1.0–1.4%). The risk of hospitalization was significantly increased in mid‐June 2020 to mid‐February 2021, while the risk of ICU admission remained stable over time. The risk of death decreased over time, with a significant drop among ≥70‐years‐olds in mid‐February 2021 to late‐June 2021; COVID‐19 vaccination started early January 2021.

**Conclusion:**

Whereas the increase in severity of Severe Acute Respiratory Syndrome Coronavirus 2 with age remained stable, the risk of death upon infection decreased over time. A significant drop in risk of death among elderly coincided with the introduction of COVID‐19 vaccination.

## 1 INTRODUCTION

The severity of Severe Acute Respiratory Syndrome Coronavirus 2 (SARS‐CoV‐2) infection—defined as the proportion of infections resulting in hospitalization, intensive care unit (ICU) admission, and death—is an important indicator to guide pandemic response measures and hospital capacity planning. These severity estimates could vary across epidemic periods due to changes in stress on the healthcare system, availability of curative treatments and vaccines, and the emergence of new virus variants of concern (VOC). The overall severity depends also on the affected population, for instance, on the distribution of infections across age groups. This emphasizes the need for severity estimates specified by age group and over different epidemic periods.

To reliably evaluate the severity of infection at the population level, representative serological surveys are needed. Several systematic reviews present serology‐based infection fatality rates (IFRs) by age, geographic region, and period.^1^, ^2^ Few European studies provide estimates of the infection hospitalization rate (IHR) and/or infection ICU admission rate (IICUR), while these indicators are arguably more relevant to policy makers. Available studies are limited to periods when the wild‐type virus was dominant and vaccination had not been introduced^3^, ^4^, ^5^ or used models to estimate the numbers of infections.^6^, ^7^, ^8^

Here, we report age‐specific estimates of the IHR, IICUR, and IFR of SARS‐CoV‐2 in the Netherlands in three epidemic periods in the period February 2020 to June 2021, based on a series of large nationwide representative serology surveys. The epidemic periods approximately cover the first wave, the second wave, and the third wave in the Netherlands, the latter of which dominated by Alpha VOC.^9^ We inferred severity estimates by combining estimates of infections with nationwide data on SARS‐CoV‐2‐positive hospitalizations and ICU admissions from a high‐quality hospital registry and with nationwide all‐cause excess deaths estimates. We explicitly accounted for reinfections and, as the roll‐out of the COVID‐19 vaccination program in the Netherlands started in January 2021, for breakthrough infections.

## 2 METHODS

### 2.1 SARS‐CoV‐2 infections

The numbers of SARS‐CoV‐2 infections by age group (<10, 10–19, …, ≥80 years) per epidemic period were based on the nationwide longitudinal serology study PIENTER Corona. Participants were selected from the Dutch population registry in an age‐ and region‐specific manner (details in literature^10^, ^11^). The study involved the collection of self‐collected fingerstick blood samples and questionnaires (including questions on general characteristics, SARS‐CoV‐2 test confirmations, and vaccination status) from the same group of participants aged 1–92 years at multiple time points. The sampling rounds used in this study were conducted in April 2020 (2637 samples), June 2020 (6813 samples), September 2020 (6093 samples), February 2021 (5981 samples), and June 2021 (5335 samples). The increase in samples from April 2020 to June 2020 follows an expansion of the cohort in order to increase the power and geographical distribution across the country.^11^

Seropositivity for SARS‐CoV‐2 was determined using a validated immunoassay that measured serum IgG antibodies against the Spike‐S1 antigen.^12^ To identify reinfections, seropositive individuals who had a fourfold increase in antibody concentration against the Spike‐S1 antigen in subsequent rounds and a negative self‐reported vaccination status (only for the rounds of mid‐February 2021 and late‐June 2021) were classified as reinfected. Among Spike S1 seropositive individuals who reported being vaccinated in subsequent rounds, a breakthrough infection was considered if they had a self‐reported SARS‐CoV‐2 polymerase chain reaction (PCR) test confirmation, seropositivity for the nucleocapsid antigen (N), a target‐protein not present in COVID‐19 vaccines, or a fourfold increase in anti‐N concentration already positive.^13^

We used the serological study rounds of June 2020 (median sampling date: June 15, 2020), February 2021 (median sampling date: February 17, 2021), and June 2021 (median sampling date: June 23, 2021) to define three epidemic periods, approximately covering infections between late‐February 2020 and mid‐June 2020 (period 1), between mid‐June 2020 and mid‐February 2021 (period 2), and between mid‐February 2021 and late‐June 2021 (period 3). Participants were excluded from the analysis for period 2 if they missed the late‐September 2020 and/or mid‐February 2021 sampling, unless they were seronegative in period 1 and seronegative (i.e., infected) in the September 2020 or February 2021 survey. Also, seropositive participants in the survey of September 2020 without a sample in period 1 were excluded as they could have been infected already in period 1. Participants were excluded from the analysis for period 3 if they missed the February 2021 or June 2021 sampling.

We estimated the age‐specific proportions infected (including reinfections and breakthrough infections) per period and their 95% confidence intervals (95% CIs), controlling for the survey design and weighting to match the distribution of the general Dutch population (as of January 1, 2020) regarding sex, age, ethnic background, and degree of urbanization. We also accounted for test specifics.^11^ Self‐reported positive SARS‐CoV‐2 PCR test confirmations for seronegative individuals were not considered, as this would interfere with the adjustment for test specifics. The age‐specific proportion infected was converted to actual numbers of infections using population data of January 1, 2020.^14^

### 2.2 SARS‐CoV‐2 hospitalizations and ICU admissions

Age‐specific SARS‐CoV‐2‐positive hospital admissions (<10, 10–19, …, ≥80 years) and ICU admissions (20–29, 30–39, …, ≥80 years) by date of admission were obtained from the National Intensive Care Evaluation COVID‐19 registry (details in Dongelmans et al.^15^). This registry contains information on all hospitalized persons with a positive SARS‐CoV‐2 test or computed tomography‐confirmed COVID‐19 for nearly all hospitals and all ICUs in the Netherlands. We included admissions since February 28, 2020.

### 2.3 Excess all‐cause death estimates

Weekly death registrations (from Thursday to Wednesday) from Statistics Netherlands^16^ by age group (<50, 50–59, 60–69, 70–79, and ≥80 years) were used to estimate all‐cause excess deaths. We fitted a Gaussian linear model to the baseline number of deaths per week over the past 5 years, including a linear trend for the long‐term effect and harmonic terms for seasonal effects.^17^ If the observed number of deaths exceeded the 97.5% upper bound of the baseline fit in a certain week, we obtained the number of excess deaths by subtracting the baseline realizations from the observed number of deaths and, otherwise, set the number of excess deaths to zero. We did not include excess deaths in the months July and August of 2020, as the numbers of confirmed COVID‐19 cases, hospitalizations, and deaths were very low in that period,^9^ and to avoid the inclusion of excess deaths from a heat wave in August 2020.

### 2.4 Statistical analysis

Due to differences in delay time between infection and seroconversion and between infection and hospitalization, ICU admission, or death, we aligned all outcomes to the symptom onset date (see Supporting Information S1, Methods for specific delay times used). The serum sampling dates of the serological study rounds in June 2020, February 2021, and June 2021 were used for defining the end dates of each epidemic period, and the day after this serum sampling date was used as the start date of the next period. For the first period, we used February 27, 2020 (first confirmed SARS‐CoV‐2 infection in the Netherlands), as the start date. Daily numbers of hospitalizations and ICU admission were then cumulated between the start and end dates of each period, as well as weekly estimates of excess deaths between the start and end weeks in which these dates fell. Point estimates of severity (IHR, IICUR, and IFR) were obtained by dividing the aligned number of outcomes (reported hospitalizations, reported ICU admissions, and mean estimates of excess deaths using 1000 simulations) by the estimated number of infections. The 95% CIs of severity were based on the 2.5% and 97.5% percentiles of 1000 Monte Carlo simulations (see Supporting Information S1, Methods).

## 3 RESULTS

### 3.1 Time course of outcomes

**FIGURE 1:**
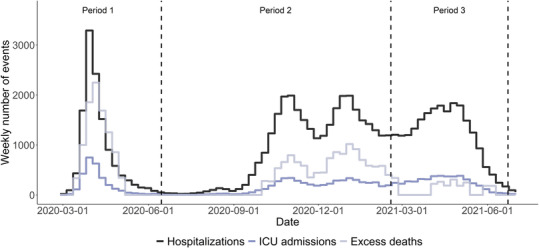
Weekly numbers of reported Severe Acute Respiratory Syndrome Coronavirus 2‐positive hospitalizations and ICU admissions by date of admission and estimated weekly number of excess deaths in the Netherlands (mean of 1000 simulations), from February 28, 2020, to July 1, 2021. The dashed lines represent the timing of the second sample round in mid‐June 2020, fourth sampling round in mid‐February 2021, and fifth sampling round in late‐June 2021 of the serological study. These surveys were used to distinguish three epidemic periods. ICU, intensive care unit.

The time course of the outcome measures together with the median sampling dates of the used serological study rounds shows that the period 1 captures the first wave, the period 2 the second wave with two consecutive peaks, and period 3 the third wave which was dominated by Alpha VOC (Figure 1). The sampling rounds at the end of the periods 1 and 3 were conducted when the epidemics had subsided to low numbers of severe events, while the sampling round at the end of period 2 was in between two periods of infection but at a high level of severe events. The curves of hospitalizations, ICU admissions, and excess deaths differ in magnitude but follow a similar pattern across periods 1 and 2. In the beginning of the period 3, the curve of excess deaths decreases faster than the other two curves, as hospitalizations were still high but no or limited excess deaths were detected. Figure S1 provides more insight in the estimated number of excess deaths per week by age group.

### 3.2 Time course of age‐specific SARS‐CoV‐2 infections

**FIGURE 2:**
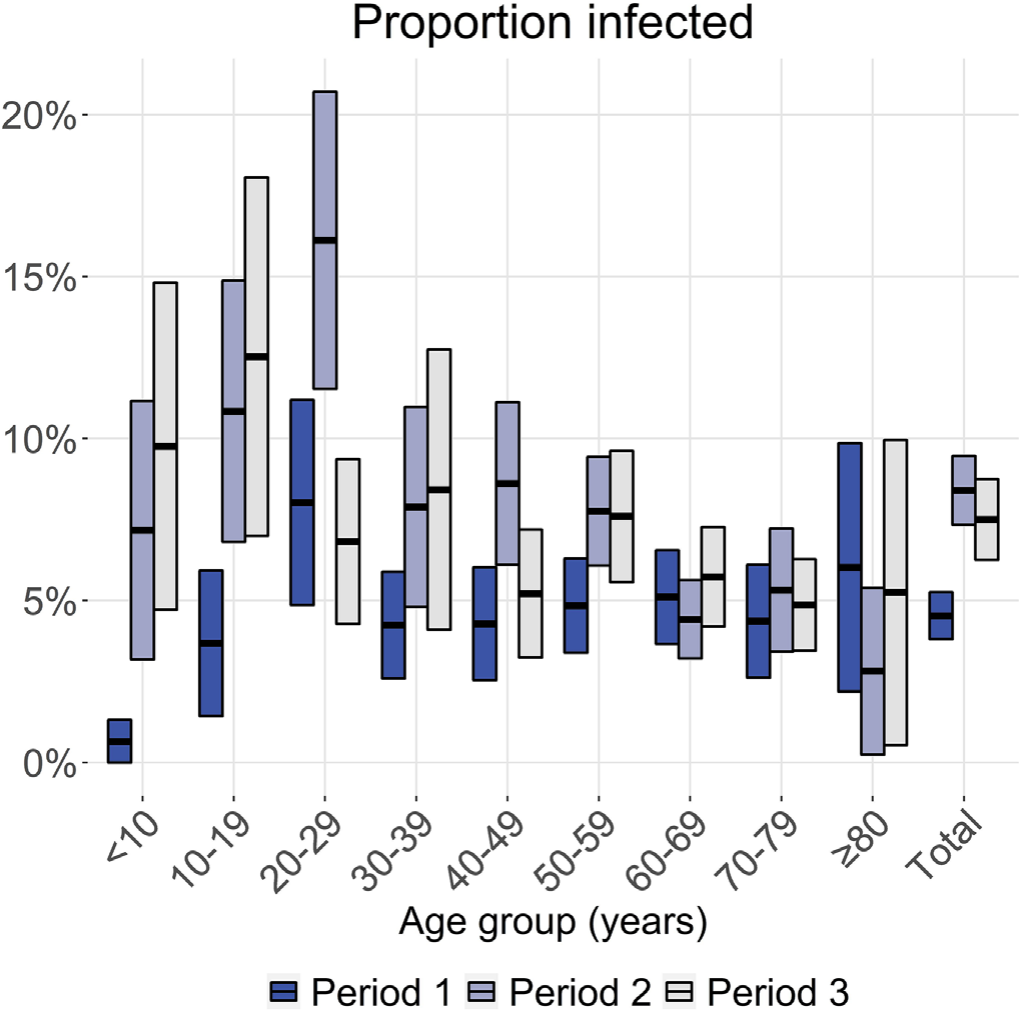
Proportion and 95% confidence interval of the study population newly infected per epidemic period, by age group, and overall. Period 1 approximately covers infections from late‐February 2020 to mid‐June 2020, period 2 from mid‐June 2020 to mid‐February 2021, and period 3 from mid‐February 2021 to late‐June 2021. Absolute numbers are available in Tables S1–S3.

Across all age groups, the estimated proportion of the survey population that experienced a new SARS‐CoV‐2 infection was 4.5% (95% CI: 3.8–5.2%) in period 1, 8.4% (95% CI: 7.3–9.5%) in period 2, and 7.5% (95% CI: 6.3–8.7%) in period 3 (Figure 2 and Tables S1–S3). On the total Dutch population of 17.4 million, this corresponds to 0.8 million, 1.5 million, and 1.2 million infections in each period, respectively. The highest proportions of infected persons were observed among 20‐ to 29‐year‐olds up to period 2 and among <20‐year‐olds in period 3. Only a small number of infections were measured among children aged below 10 years in period 1.

### 3.3 Hospitalization and ICU admission per infection

**FIGURE 3:**
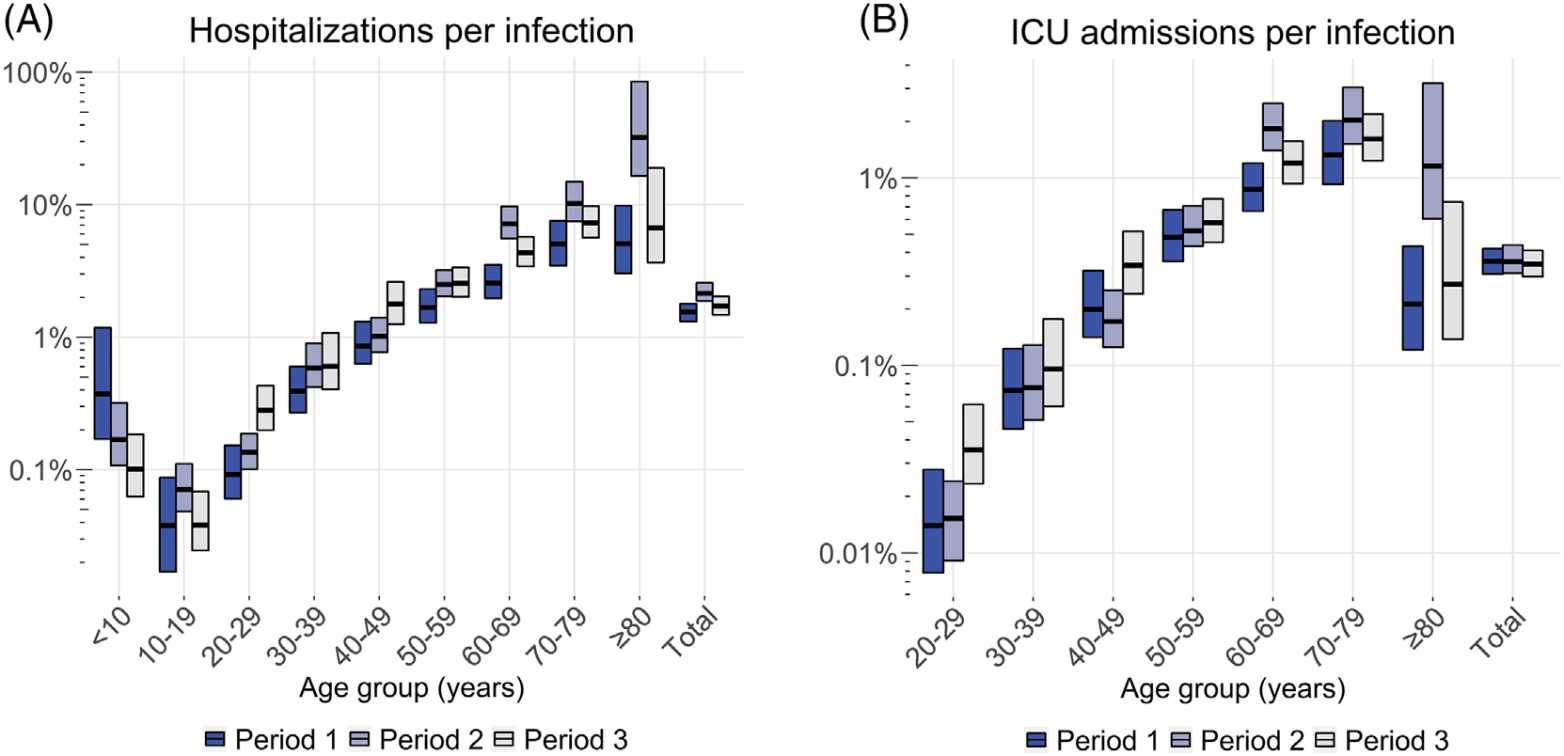
Estimated age‐specific and overall infection hospitalization rates (A) and infection ICU admission rates (B) in the Netherlands per epidemic period. Horizontal lines represent the point estimates and bars the 95% ranges using 1000 simulations. Period 1 approximately covers infections from late‐February 2020 to mid‐June 2020, period 2 from mid‐June 2020 to mid‐February 2021, and period 3 from mid‐February 2021 to late‐June 2021. Note that the *y*‐axes use a log‐scale. Absolute estimates are available in the Tables S1–S3. ICU, intensive care unit.

The overall number of SARS‐CoV‐2 positive registered hospitalizations was 12,220 in period 1, 31,382 in period 2, and 22,471 in period 3. Divided by the estimated numbers of infections, these result in IHRs of 1.5% (95% CI: 1.3–1.8%), 2.1% (95% CI: 1.9–2.6%), and 1.7% (95% CI: 1.5–2.0%), respectively (Figure 3A and Tables S1–S3); hence, the overall IHR was statistically significantly increased in period 2 compared with period 1, but not statistically significantly increased in period 3 compared with period 1. Among these hospitalizations, there were 2836 ICU admissions in period 1, 5230 in period 2, and 4537 in period 3, resulting in IICURs of 0.36% (95% CI: 0.31–0.42%), 0.36% (95% CI: 0.31–0.44%), and 0.35% (95% CI: 0.30–0.41%), respectively (Figure 3B and Tables S1–S3); hence, the overall IICUR remained stable over time. In each period, the IHR and IICUR show a nearly consistent pattern of an accelerating, almost exponential, increase with increasing age in the age range 20–29 to 70–79 years. The IHR was higher among 0‐ to 9‐year‐olds than 10‐ to 19‐year‐olds and similar between 70‐ to 79‐year‐olds and ≥80‐year‐olds. The IICUR was lower among ≥80‐year‐olds compared with 70‐ to 79‐year‐olds. Over the whole study period, the IHR doubled with every 9.0 years of increase in age, and the IICUR doubled faster, with every 7.5 years of increase in age. When comparing across epidemic periods, the IHR and IICUR among 20‐ to 59‐year‐olds were the highest in period 3, although not statistically significantly different compared with periods 1 and 2 for most age groups within this age range. Among ≥60‐year‐olds, highest IHR and IICUR were found in the period 2.

### 3.4 Excess deaths per infection

**FIGURE 4:**
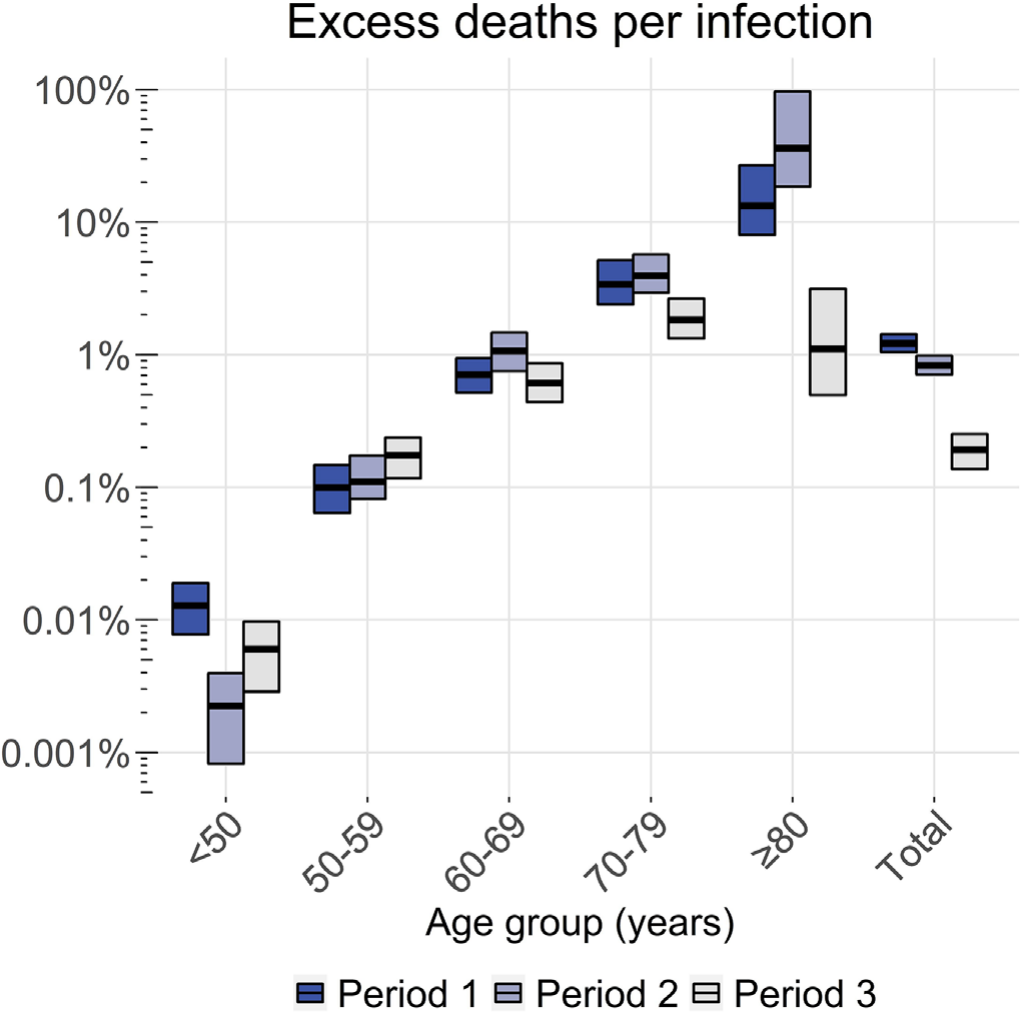
Estimated overall and age‐specific infection fatality rate in the Netherlands per epidemic period. Horizontal lines represent the point estimates and bars the 95% ranges using 1000 simulations. Period 1 approximately covers infections from late‐February 2020 to mid‐June 2020, period 2 from mid‐June 2020 to mid‐February 2021, and period 3 from mid‐February 2021 to late‐June 2021. Note that the *y*‐axis uses a log‐scale. Absolute numbers are available in Tables S1–S3.

The average total number of excess deaths was estimated at 9585 (95% CI: 9062–10,112) in period 1, 12,223 (95% CI: 11,266–13,298) in period 2, and 2512 (95% CI: 1897–3147) in period 3, resulting in overall IFRs of 1.2% (95% CI: 1.0–1.4%), 0.83% (95% CI: 0.71–0.98%), and 0.19% (95% CI: 0.14–0.25%), respectively; hence, the overall IFR significantly decreased over time. The IFR increased exponentially with age in each period (Figure 4 and Tables S1–S3), except in period 3, where the IFR tends to drop among 60‐ to 69‐year‐olds and significantly drops among 70‐ to 79‐year‐olds and, particularly, among ≥80‐year‐olds. Across the whole study period, the IFR doubled with every 4.3 years of increase of age above the age of 50.

## 4 DISCUSSION

We analyzed the age‐specific severity of SARS‐CoV‐2 in the Netherlands across three epidemic periods in the period February 2020 to June 2021. These periods approximately corresponded to the first wave, the second wave, and the third wave, which was dominated by VOC alpha. In each of these three periods, we found a consistent accelerating, almost exponential, increase of severity with age in the age range 10–79 years, and this rate of increase in severity with age being determined by the severity of the outcome: the highest for death, intermediate for ICU admission, and the lowest for hospitalization. The IFR dropped significantly among ≥70‐years‐olds in the third period.

While the IFR increased with age up to ≥80‐year‐olds in the first two epidemic periods, the IICUR increased up to 70–79‐year‐olds and then decreased among ≥80‐year‐olds. This divergence in trends between the IFR and the IICUR in elderly could be specific to the Netherlands, where frail elderly are often—after consultation with their general practitioner and family—reluctant to be admitted to the ICU in particular, as ICU treatment may be considered too invasive.^18^ During the COVID‐19 pandemic, this reluctance may have been stronger due to the high demand for hospital care. Given that a large share of the mortality occurred in the elderly, this presumably explains also why the overall IFR is more than three times higher than the overall IICUR in the first period and more than two times higher in the second period.

The overall IFR decreased over time. From the first to the second period, the decrease in overall IFR was not accompanied with a decrease in age‐specific IFRs, meaning that the overall decrease was mainly driven by a shift of infections to younger age groups instead of an actual decrease in risk of death. From the second to the third period, the drop in overall IFR was mainly driven by a substantial decrease in IFR among 80‐year‐olds, which coincides with the roll‐out of the vaccination program in this age group. The COVID‐19 vaccination program in the Netherlands started 6 weeks prior to the start of the third period, with prioritization of the elderly population (beginning with the oldest individuals) and health care workers. By early‐March 2021, at least 70% of individuals aged ≥80 years had received their initial vaccine dose, and by mid‐April 2021, at least 70% of this age group had been fully vaccinated (Figure S2, details in National Institute for Public Health and the Environment^9^). Moreover, the vaccination coverage among 70‐ to 79‐year‐olds, for whom the IFR was also significantly decreased in the third period, reached a minimum of 70% for the first dose by mid‐April 2021 and at least 70% for the complete vaccine series by late‐May 2021. For 60‐ to 69‐year‐olds, who exhibited a tendency for decreased IFR in the third period, at least 70% coverage for the first dose was achieved by early‐May 2021. The coverage for the complete vaccine series among this age group was reached by end‐June 2021, after the end of the third period.

The decrease in overall IFR over time was not accompanied with a decrease in overall IICUR or in overall IHR; the IHR was even significantly increased in the second period compared with the first period. A possible explanation could be that the maximum capacities in hospitals in the Netherlands were reached during the first period, and a reduction in pressure on bed capacities in following periods resulted in easing of the admission policy in the second period. From the second period onward, treatments with dexamethasone and tocilizumab became available, as well as different forms of oxygen administration, even at home.^19^ This led to a change in patient characteristics of ICU‐admitted patients, being on average older and having more comorbidities in the second period than in the first period.^15^ In the third period, vaccination presumably had more impact on the IFR than on the IHR/IICUR, because a decent share of COVID‐19‐associated deaths occurred among nursing homes residents, who were prioritized for vaccination but often not admitted to the hospital.

Although not statistically significant, the IHR and IICUR among 20–59‐years‐olds tend to be higher in the third period compared with previous periods. This increase might be related to the emergence of the Alpha VOC, which became the dominant circulating strain in the third period. The Alpha VOC is associated with an increased severity among community‐tested SARS‐CoV‐2 cases as compared with wild‐type SARS‐CoV‐2 in England^20^ and Denmark.^21^ The relatively higher severity estimates among ≥60‐year‐olds in the second period may be explained by an underestimate of infections in this age range, as the number of infections in that period was close to the number of COVID‐19 notifications,^9^ while in the other periods, the estimated numbers of infections among ≥60‐year‐olds were substantially higher than the numbers of COVID‐19 notifications.

Comparing our all‐cause excess deaths estimates with confirmed or suspected COVID‐19 deaths from cause‐of‐death certificates,^22^ we find the mean number of excess deaths to be 5% lower in the first period (9.6 thousand vs. 10.1 thousand), 24% lower in the second period (12.2 thousand vs. 16.0 thousand), and 54% lower in the third period (2.5 thousand vs. 5.4 thousand). Excess mortality is an indirect measure of COVID‐19 mortality that could be affected by changes in mortality from other causes. Pandemic response measures have also resulted in a large reduction in deaths from other respiratory pathogens during the winter of 2020–2021, like from influenza,^9^ potentially explaining why we found lower excess mortality than the numbers of COVID‐19 deaths from cause‐of‐death statistics in the second and third periods. An advantage of the use of excess death mortality is its standardization, allowing international comparisons, and its shorter delay in data availability compared with cause‐of‐death certificates.

The estimated overall IFR of 1.2% in the Netherlands in the first period compares well with findings from other Western‐European countries, estimating the IFR in the range of 0.5% to 1.8% for the same period.^2^, ^4^, ^23^, ^24^, ^25^ Our finding of a decline in overall IFR from the first to the second period explained by a different age distribution of infections was also seen in Norway.^4^ The overall, and age‐specific, estimates of IHR in the Netherlands tend to be lower than in other European countries, including France, Denmark, and Norway,^3^, ^4^, ^5^ particularly among the elderly. The international comparability of IHR may be harder than IFR due to differences in hospital capacities and admission criteria. Our finding that the increase in severity with age was faster for more severe outcomes was supported by a study from France^3^ and by an international meta‐analysis.^26^

The study has several limitations. First, despite weighting the serological study sample, there could be nonparticipation of certain groups that had an increased risk of infection, such as ethnic minority groups^27^ or nursing home residents.^28^ Also, participants of surveys may adhere better to pandemic response measures than the general population. Second, a small proportion of infected persons does not mount detectable antibody titers,^29^ although we used a highly accurate serological assay and corrected the seroprevalence estimates for a loss in sensitivity. Third, we could have missed reinfections or breakthrough infections among individuals who did not exhibit a fourfold increase in concentration between successive study rounds. Furthermore, vaccinated individuals might seroconvert slightly less to anti‐N than unvaccinated individuals,^30^ potentially resulting in the omission of some breakthrough infections. Nevertheless, given that reinfections and breakthrough infections comprise a minor fraction of the total number of infections during our study period and SARS‐CoV‐2 test results were taken into account for vaccinated persons too, we do not anticipate significant impact on the severity outcomes. Fourth, an unknown proportion of the reported SARS‐CoV‐2‐positive hospitalizations was not admitted due to COVID‐19 but incidentally testing positive for SARS‐CoV‐2 at admission, which may have led to an overestimation of COVID‐19‐related hospitalizations and ICU admissions, and, consequently, of severity. However, as regular care was scaled down in the Netherlands during most of the study period, we expect this would concern only a minority of admitted patients.

A major strength of our study is the use of longitudinal serology data from a large, population‐based cohort across all ages instead of relying on the (usually lower) numbers of COVID‐19 notifications. Additionally, the study rounds were conducted at frequent intervals, thereby minimizing the potential for missing infections caused by waning immunity. Data from a high‐quality national hospital‐based COVID‐19 registry provided us with complete data on hospitalizations and ICU admissions. We accounted for imperfect serological testing, differences in delay times to events, reinfections, and breakthrough infections. The findings cover a range of outcomes, a long time period, and the full age range. It is consistent with previously reported severity estimates for a limited period, a limited age range, or a single outcome for other European countries. It can be used to interpolate these findings and extrapolate them to other outcomes, periods, and age groups across a range of countries.

## 5 CONCLUSION

Infection‐based estimates of severity of SARS‐CoV‐2 reveal a nearly consistent pattern of increased severity of SARS‐CoV‐2 with increasing age across the first three epidemic periods in the Netherlands. We confirm that the increase in severity with age was faster for more severe outcomes. Differences within age groups between periods might be explained by different circulating SARS‐CoV‐2 variants and the introduction of SARS‐CoV‐2 vaccination. We demonstrate that combining serological data from a longitudinal, nationwide survey with the numbers of severe events from hospital and death registries can provide useful insights in monitoring changes in severity of SARS‐CoV‐2 infection over time. Use of excess mortality for monitoring the IFR of SARS‐CoV‐2 appears to be challenging in periods with deviating numbers of deaths from other causes, for instance, during weeks with extreme temperatures or due to effects of pandemic response measures on other infectious diseases.

## AUTHOR CONTRIBUTIONS

The project was conceptualized by PTdB, JvdK, ERAV, LvA, EG, SvdH, and JW. Input in different parts of data collection and analysis: (i) seroprevalences: ERAV, GdH, FvdK, and HEdM; (ii) hospitalizations/ICU admissions: DD, DdL, and RIVM COVID‐19 surveillance and epidemiology team; (iii) excess mortality: JvdK, LvA, and LS; and (iv) Laboratory‐confirmed cases: RIVM COVID‐19 surveillance and epidemiology team. JvdK developed the model code, which was revised by PTdB. The initial draft of the manuscript was written by PTdB, which was critically revised by JvdK, ERAV, LvA, DD, ABvGL, GdH, AH, FvdK, DdL, LS, HEdM, EG, SvdH, and JW.

## CONFLICT OF INTEREST STATEMENT

None.

## FUNDING INFORMATION

This work was supported by the Netherlands Ministry of Health, Welfare and Sport. This project has received funding from the European Union's Horizon 2020 research and innovation program—project EpiPose (grant agreement number 101003688).

## ETHICS STATEMENT

Not required.

### PEER REVIEW

The peer review history for this article is available at https://www.webofscience.com/api/gateway/wos/peer-review/10.1111/irv.13174.

## RIVM COVID‐19 SURVEILLANCE AND EPIDEMIOLOGY TEAM

The members of the RIVM COVID‐19 surveillance and epidemiology team are as follows: Agnetha Hofhuis, Anne Teirlinck, Anne‐Wil Valk, Carolien Verstraten, Claudia Laarman, Cheyenne van Hagen, Femke Jongenotter, Fleur Petit, Guido Willekens, Irene Veldhuijzen, Jan van de Kassteele, Janneke van Heereveld, Janneke Heijne, Kirsten Bulsink, Liselotte van Asten, Liz Jenniskens, Lieke Wielders, Loes Soetens, Maarten Mulder, Maarten Schipper, Marit de Lange, Naomi Smorenburg, Nienke Neppelenbroek, Patrick van den Berg, Priscila de Oliveira Bressane Lima, Rolina van Gaalen, Steven Nijman, Senna van Iersel, Stijn Andeweg, Susan Lanooij, Tara Smit, Thomas Dalhuisen, Don Klinkenberg, Jantien Backer, Pieter de Boer, Scott McDonald, Amber Maxwell, Annabel Niessen, Brechje de Gier, Danytza Berry, Daphne van Wees, Dimphey van Meijeren, Eric R.A. Vos, Frederika Dijkstra, Jeanet Kemmeren, Kylie Ainslie, Marit Middeldorp, Mirjam Knol, Albert Jan van Hoek, Hester de Melker, Jacco Wallinga, Rianne van Gageldonk‐Lafeber, Susan Hahné, and Susan van den Hof.

## Supporting information


**Figure S1:** Weekly numbers of all‐cause deaths (black line) in the Netherlands by age‐group and in the total population as well as the baseline fit with the 95%‐prediction interval (blue colored area) and the center of the interval (blue line). Panel A shows the data and fit from July 2015 to June 2020, which was used for excess deaths estimation in the first epidemic period (late‐February 2020 – mid‐June 2020), and Panel B shows the data and fit from July 2016 to June 2021, which was used for the estimation of excess deaths in the second epidemic period (mid‐June 2020 – mid‐February 2021) and the third epidemic period (mid‐February 2021 – late‐June 2021).
**Table S1:** Outcomes and severity estimates of the first epidemic period, from late‐February 2020 to mid‐June 2020.
**Table S2:** Outcomes and severity estimates of the second epidemic period, from mid‐June 2020 to mid‐February 2021.
**Table S3:** Outcomes and severity estimates of the third epidemic period, mid‐February 2021 to late‐June 2021.
**Figure S2:** The vaccination coverage of the COVID‐19 vaccination programme in the Netherlands over time and by age‐group between January 2021 and December 2021. Panel A shows the vaccination coverage of at least one dose, and panel B for the complete vaccination series. Figures are adapted from [2].

## Data Availability

All data produced in the present study are available upon reasonable request to the authors.
