# Supplementary material for: Age‐specific severity of severe acute respiratory syndrome coronavirus 2 in February 2020 to June 2021 in the Netherlands
Source: Influenza Other Respir Viruses. 2023 Aug 22;17(8):e13174. doi: 10.1111/irv.13174 (PMC10444602; doi:10.1111/irv.13174)
Supplement: Supplementary file 1 — Figure S1: Weekly numbers of all‐cause deaths (black line) in the Netherlands by age‐group and in the total population as well as the baseline fit with the 95%‐prediction interval (blue colored area) and the center of the interval (blue line). Panel A shows the data and fit from July 2015 to June 2020, which was used for excess deaths estimation in the first epidemic period (late‐February 2020 – mid‐June 2020), and Panel B shows the data and fit from July 2016 to June 2021, which was used for the estimation of excess deaths in the second epidemic period (mid‐June 2020 – mid‐February 2021) and the third epidemic period (mid‐February 2021 – late‐June 2021). Table S1: Outcomes and severity estimates of the first epidemic period, from late‐February 2020 to mid‐June 2020. Table S2: Outcomes and severity estimates of the second epidemic period, from mid‐June 2020 to mid‐February 2021. Table S3: Outcomes and severity estimates of the third epidemic period, mid‐February 2021 to late‐June 2021. Figure S2: The vaccination coverage of the COVID‐19 vaccination programme in the Netherlands over time and by age‐group between January 2021 and December 2021. Panel A shows the vaccination coverage of at least one dose, and panel B for the complete vaccination series. Figures are adapted from [2]. [file IRV-17-e13174-s001.docx]

# Supplemental materials

## Manuscript title:

Age-specific severity of Severe Acute Respiratory Syndrome Coronavirus 2 in February 2020 to June 2021 in the Netherlands

## Authors:

Pieter T. de Boer^1^, Jan van de Kassteele^1*^, Eric R.A. Vos^1*^, Liselotte van Asten^1^, Dave A. Dongelmans^2,3^, Arianne B. van Gageldonk-Lafeber^1^, Gerco den Hartog^1,4^, Agnetha Hofhuis^1^, Fiona van der Klis^1^, Dylan W. de Lange^5^, Lenny Stoeldraijer^6^, the RIVM COVID-19 epidemiology and surveillance team^1^, Hester E. de Melker^1^, Eveline Geubbels^1^, Susan van den Hof^1*^, Jacco Wallinga^1,7*^

## Affiliations:

1. Center for Infectious Disease Control, National Institute for Public Health and the Environment (RIVM), Bilthoven, the Netherlands
2. Department of Intensive Care Medicine, Amsterdam UMC (location AMC), Amsterdam, the Netherlands.
3. Amsterdam Public Health Research Institute, Amsterdam, the Netherlands.
4. Laboratory of Medical Immunology, Radboudumc, Nijmegen, the Netherlands
5. Intensive Care, University Medical Center Utrecht, University Utrecht, Utrecht, the Netherlands.
6. Statistics Netherlands, the Hague, the Netherlands
7. Department of Biomedical Datasciences, Leiden University Medical Center, Leiden, the Netherlands.

*These authors contributed equally to the manuscript

## Supplemental methods

### Excess mortality estimates

To estimate numbers of excess deaths by age-group, we utilized age-specific national death registration data (<50y, 50-60y, 70-80y and 80+y) from Statistics Netherlands. We obtained weekly numbers, starting from Thursday and ending on Wednesday) for the period July 1, 2015 to 15 September, 2021. Definitive numbers Monday–Sunday in different age-categories are publicly available at <https://opendata.cbs.nl>.

For estimation, we employed Gaussian linear regression models with a linear and periodic time trend similar to that described by Van Asten et al [1]. For estimation, we employed Gaussian linear regression models with a linear and periodic time trend similar to that described by Van Asten et al. The past 5-year data were used to estimate the current weekly baseline number of deaths. Periods with high deaths in winter (25% of highest observations) and summer (20% of the highest observations in July and August) were removed to avoid baselines being influenced by previous events such as outbreaks (e.g., influenza epidemics before March 2020 or the SARS-CoV-2 outbreak since March 2020 for later epidemic waves) or extreme temperatures (e.g., heat waves and extreme cold). Baselines are updated annually by the first week of July; for the first epidemic period we used data starting from July 2015, and for the second and third epidemic periods we used data from July 2016 onwards. Weeks in which the number of deaths exceeds the upper 97.5% upper bound of the baseline fit are classified as weeks with excess deaths. The excess deaths for those weeks are calculated by subtracting the center of the baseline fit from the observed number of deaths. Excess deaths during the months July 2020 and August 2020 were excluded, as the number of reported SARS-CoV-2-positive cases, COVID-19 hospitalizations and COVID-19 deaths in those months were very low in those months, and to avoid the inclusion of excess deaths from a heatwave in early August of 2020.

### Confidence intervals of severity estimates

Confidence intervals of severity estimates were based on 1,000 Monte Carlo simulations. We generated 1,000 Monte Carlo realizations of age-specific proportions infected per epidemic period by sampling from a beta-distribution, and for each sampled proportion we generated the number of infections by sampling from a Binomial distribution using the national population sizes of 1 January 2020. We fitted a negative Binomial generalized additive model to the daily number of hospitalizations and ICU admissions using a penalized spline to account for the date effect and day-of-the-week effects, and the fitted model was used to generate 1,000 Monte Carlo realizations from the predictive distributions for hospitalizations and ICU admissions. These realizations include both the uncertainty of the parameter estimates as well as residual error (resulting from a similar sampling process as with the number of infections). We generated 1,000 Monte Carlo realizations of excess deaths from the predictive distribution of the baseline mortality, conditional to the fitted Gaussian linear model. For each epidemic period, we sampled 1000 Monte Carlo realizations of the end-date from the empirical distribution of the serological sampling date, with a median of 15 June 2020 (Interquartile Range [IQR]: 11 June 2020 – 17 June 2020) for the first period, a median of 17 February 2021 (IQR: 15 February 2021 – 19 February 2021) for the second period, and a median of 23 June 2021 (IQR: 21 June 2021 – 28 June 2021) for the third period. The start dates of the second and third period were set at one day after the sampled end-dates of the previous period, and fixated at 27 February 2020 for the first period (first confirmed SARS-CoV-2 infection in the Netherlands). To align all outcomes to the symptom onset date, we sampled 1,000 realizations of the delay time between symptom onset and seroconversion from a gamma distribution, with a median of 12 days (IQR: 9-14). A 1,000 Monte Carlo realizations of delay times from symptom onset to hospitalization, ICU admission and death were sampled from empirical distributions, using data of laboratory-confirmed cases included in the Dutch national COVID-19 notification database up to 1 July 2021, and for whom the symptom onset date was available. These median delays were 5 days (IQR: 2-9 days) from symptom onset to hospitalization, 7 days (IQR: 4-11 days) from symptom onset to ICU admission, and 11 days (IQR: 7-15 days) from symptom onset to death. Interval estimates were obtained by dividing the 1,000 Monte Carlo realizations of the sampled cumulative number of aligned outcomes by the sampled number of infections, taking the 2.5% and 97.5% percentiles of the Monte Carlo realizations as the 95% CI.

## Supplemental results

**A**


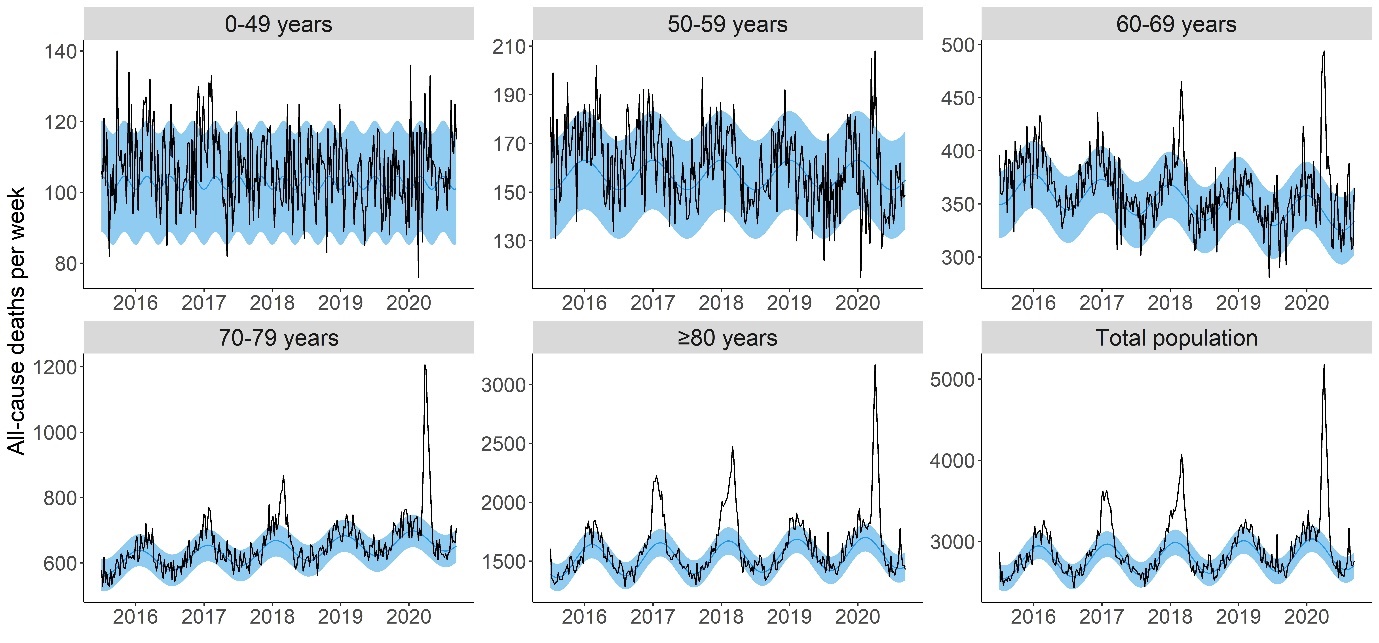


**B**

**
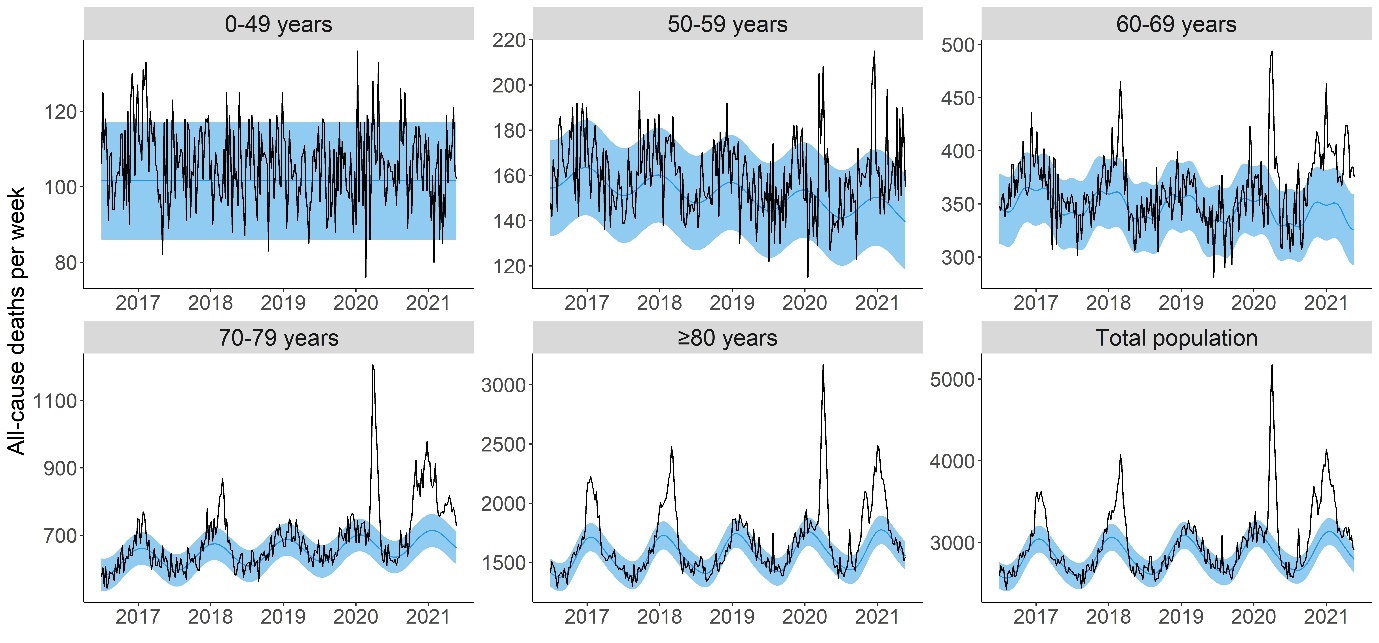
**

Figure S1: Weekly numbers of all-cause deaths (black line) in the Netherlands by age-group and in the total population as well as the baseline fit with the 95%-prediction interval (blue colored area) and the center of the interval (blue line). Panel A shows the data and fit from July 2015 to June 2020, which was used for excess deaths estimation in the first epidemic period (late-February 2020 – mid-June 2020), and Panel B shows the data and fit from July 2016 to June 2021, which was used for the estimation of excess deaths in the second epidemic period (mid-June 2020 – mid-February 2021) and the third epidemic period (mid-February 2021 – late-June 2021).

Table S1: Outcomes and severity estimates of the first epidemic period, from late-February 2020 to mid-June 2020.

| Age-group (years) | Number of samples used from the serology study | Change in SARS-CoV-2 seroprevalence (%, 95% CI) | Population size (thousands) | Estimated infections (thousands, 95% CI) | Hospital admissions | IHR (%, 95% CI) | ICU admissions | IICUR (%, 95% CI) | Estimated excess deaths (mean, 95% CI) | IFR (%, 95% CI) |
| --- | --- | --- | --- | --- | --- | --- | --- | --- | --- | --- |
| <10 | 505 | 0.65 (0.00-1.32) | 1,773 | 12 (0-23) | 43 | 0.37 (0.17-1.20) |  |  |  |  |
| 10-19 | 623 | 3.7 (1.4-5.9) | 2,002 | 74 (29-119) | 28 | 0.038 (0.017-0.087) |  |  |  |  |
| 20-29 | 698 | 8.0 (4.9-11.2) | 2,234 | 179 (109-250) | 165 | 0.092 (0.060-0.152) | 25 | 0.014 (0.0078-0.028) |  |  |
| 30-39 | 777 | 4.2 (2.6-5.9) | 2,148 | 91 (56-127) | 357 | 0.39 (0.27-0.60) | 67 | 0.074 (0.046-0.123) |  |  |
| 40-49 | 905 | 4.3 (2.5-6.0) | 2,208 | 95 (56-133) | 809 | 0.86 (0.63-1.31) | 188 | 0.20 (0.14-0.32) |  |  |
| <50 | 3,508 | 4.3 (3.4-5.2) | 10,365 | 446 (348-544) |  |  |  |  | 57 (36-77) | 0.013 (0.008-0.019) |
| 50-59 | 1,092 | 4.8 (3.4-6.3) | 2,532 | 123 (86-159) | 2,056 | 1.7 (1.3-2.3) | 590 | 0.48 (0.36-0.67) | 122 (87-159) | 0.10 (0.06-0.15) |
| 60-69 | 1,217 | 5.1 (3.7-6.6) | 2,114 | 108 (77-138) | 2,770 | 2.6 (2.0-3.5) | 938 | 0.87 (0.67-1.20) | 764 (659-869) | 0.71 (0.52-0.94) |
| 70-79 | 843 | 4.4 (2.6-6.1) | 1,574 | 69 (41-96) | 3,472 | 5.1 (3.5-7.5) | 911 | 1.3 (0.9-2.0) | 2,340 (2,171-2,503) | 3.4 (2.4-5.2) |
| ≥80 | 153 | 6.0 (2.2-9.9) | 822 | 49 (18-81) | 2,513 | 5.1 (3.0-9.8) | 105 | 0.21 (0.12-0.43) | 6,595 (6,110-7,071) | 13 (8-27) |
| Total | 6,813 | 4.5 (3.8-5.2) | 17,408 | 789 (663-914) | 12,220 | 1.5 (1.3-1.8) | 2,836 | 0.36 (0.31-0.42) | 9,585 (9,062-10,112) | 1.2 (1.0-1.4) |

CI: Confidence interval, IFR: Infection fatality rate, IHR: Infection hospitalization rate, IFR: Infection fatality rate, IICUR: infection intensive care unit admission rate

Table S2: Outcomes and severity estimates of the second epidemic period, from mid-June 2020 to mid-February 2021.

| Age-group (years) | Number of samples used from the serology study* | Change in SARS-CoV-2 seroprevalence (%, 95% CI) | Population size (thousands) | Estimated infections (thousands, 95% CI) | Hospital admissions | IHR (%, 95% CI) | ICU admissions | IICUR (%, 95% CI) | Estimated excess deaths (mean, 95% CI) | IFR (%, 95% CI) |
| --- | --- | --- | --- | --- | --- | --- | --- | --- | --- | --- |
| <10 | 274 | 7.2 (3.2-11.2) | 1,773 | 127 (56-198) | 214 | 0.17 (0.11-0.32) |  |  |  |  |
| 10-19 | 453 | 10.8 (6.8-14.8) | 2,002 | 217 (136-298) | 154 | 0.071 (0.048-0.110) |  |  |  |  |
| 20-29 | 508 | 16.1 (11.5-20.7) | 2,234 | 360 (258-462) | 488 | 0.14 (0.10-0.19) | 55 | 0.015 (0.009-0.024) |  |  |
| 30-39 | 640 | 7.9 (4.8-11.0) | 2,148 | 169 (103-236) | 994 | 0.59 (0.42-0.90) | 129 | 0.076 (0.051-0.128) |  |  |
| 40-49 | 761 | 8.6 (6.1-11.1) | 2,208 | 190 (135-246) | 1,941 | 1.0 (0.8-1.4) | 326 | 0.17 (0.13-0.25) |  |  |
| <50 | 2,636 | 10.2 (8.5-11.8) | 10,365 | 1,058 (886-1,229) |  |  |  |  | 24 (9-40) | 0.0023 (0.0008-0.0040) |
| 50-59 | 966 | 7.8 (6.1-9.4) | 2,532 | 197 (154-239) | 4,909 | 2.5 (2.0-3.2) | 1,026 | 0.52 (0.43-0.71) | 224 (174-276) | 0.11 (0.08-0.17) |
| 60-69 | 1,093 | 4.4 (3.2-5.6) | 2,114 | 93 (68-119) | 6,684 | 7.2 (5.5-9.7) | 1,709 | 1.8 (1.4-2.5) | 968 (787-1,154) | 1.1 (0.8-1.5) |
| 70-79 | 787 | 5.3 (3.4-7.2) | 1,574 | 84 (54-114) | 8,531 | 10 (7-15) | 1,708 | 2.0 (1.5-3.0) | 3,349 (3,022-3,687) | 4.0 (2.9-5.7) |
| ≥80 | 143 | 2.8 (0.3-5.4) | 822 | 23 (2-44) | 7,467 | 32 (16-85) | 268 | 1.2 (0.6-3.2) | 8,374 (7,632-9,079) | 36 (19-97) |
| Total | 5,625 | 8.4 (7.3-9.5) | 17,408 | 1,462 (1,278-1,647) | 31,382 | 2.1 (1.9-2.6) | 5,230 | 0.36 (0.31-0.44) | 12,223 (11,266-13,298) | 0.83 (0.71-0.98) |

CI: Confidence interval, IFR: Infection fatality rate, IHR: Infection hospitalization rate, IFR: Infection fatality rate, IICUR: infection intensive care unit admission rate

*The total number of samples included in the analysis is lower than the total number of samples obtained during the study round, as individuals with missing data in previous rounds were excluded.

Table S3: Outcomes and severity estimates of the third epidemic period, mid-February 2021 to late-June 2021.

| Age-group (years) | Number of samples used from the serology study* | Change in SARS-CoV-2 seroprevalence (%, 95% CI) | Population size (thousands) | Estimated infections (thousands, 95% CI) | Hospital admissions | IHR (%, 95% CI) | ICU admissions | IICUR (%, 95%CI) | Estimated excess deaths (mean, 95% CI) | IFR (%, 95% CI) |
| --- | --- | --- | --- | --- | --- | --- | --- | --- | --- | --- |
| <10 | 205 | 9.8 (4.7-14.8) | 1,773 | 173 (84-263) | 175 | 0.10 (0.06-0.18) |  |  |  |  |
| 10-19 | 387 | 12.5 (7.0-18) | 2,002 | 251 (140-362) | 96 | 0.038 (0.025-0.068) |  |  |  |  |
| 20-29 | 411 | 6.8 (4.3-9.4) | 2,234 | 152 (96-209) | 428 | 0.28 (0.20-0.43) | 54 | 0.035 (0.023-0.062) |  |  |
| 30-39 | 565 | 8.4 (4.1-12.7) | 2,148 | 181 (88-274) | 1,091 | 0.60 (0.40-1.08) | 173 | 0.096 (0.060-0.177) |  |  |
| 40-49 | 713 | 5.2 (3.2-7.2) | 2,208 | 115 (72-159) | 2,046 | 1.8 (1.3-2.6) | 393 | 0.34 (0.24-0.52) |  |  |
| <50 | 2,281 | 8.4 (6.5-10.3) | 10,365 | 873 (675-1,070) |  |  |  |  | 52 (26-80) | 0.0060 (0.0029-0.0097) |
| 50-59 | 923 | 7.6 (5.6-9.6) | 2,532 | 192 (141-244) | 4,923 | 2.6 (2.0-3.4) | 1,109 | 0.58 (0.45-0.77) | 336 (255-413) | 0.17 (0.12-0.24) |
| 60-69 | 1,032 | 5.7 (4.2-7.3) | 2,114 | 121 (89-154) | 5,263 | 4.3 (3.4-5.7) | 1,455 | 1.2 (0.9-1.6) | 744 (604-892) | 0.61 (0.44-0.86) |
| 70-79 | 806 | 4.9 (3.4-6.3) | 1,574 | 77 (54-99) | 5,567 | 7.3 (5.6-9.8) | 1,235 | 1.6 (1.2-2.2) | 1,401 (1,127-1,658) | 1.8 (1.3-2.6) |
| ≥80 | 149 | 5.3 (0.54-10.0) | 822 | 43 (4-82) | 2,882 | 6.7 (3.7-18.9) | 117 | 0.27 (0.14-0.74) | 477 (306-652) | 1.1 (0.5-3.1) |
| Total | 5,191 | 7.5 (6.3-8.7) | 17,408 | 1,306 (1,090-1,523) | 22,471 | 1.7 (1.5-2.0) | 4,537 | 0.35 (0.30-0.41) | 2,512 (1,897-3,147) | 0.19 (0.14-0.25) |

CI: Confidence interval, IFR: Infection fatality rate, IHR: Infection hospitalization rate, IFR: Infection fatality rate, IICUR: infection intensive care unit admission rate.
*The total number of samples included in the analysis is lower than the total number of samples obtained during the study round, as individuals with missing data in previous rounds were excluded.

## Supplemental discussion

**A

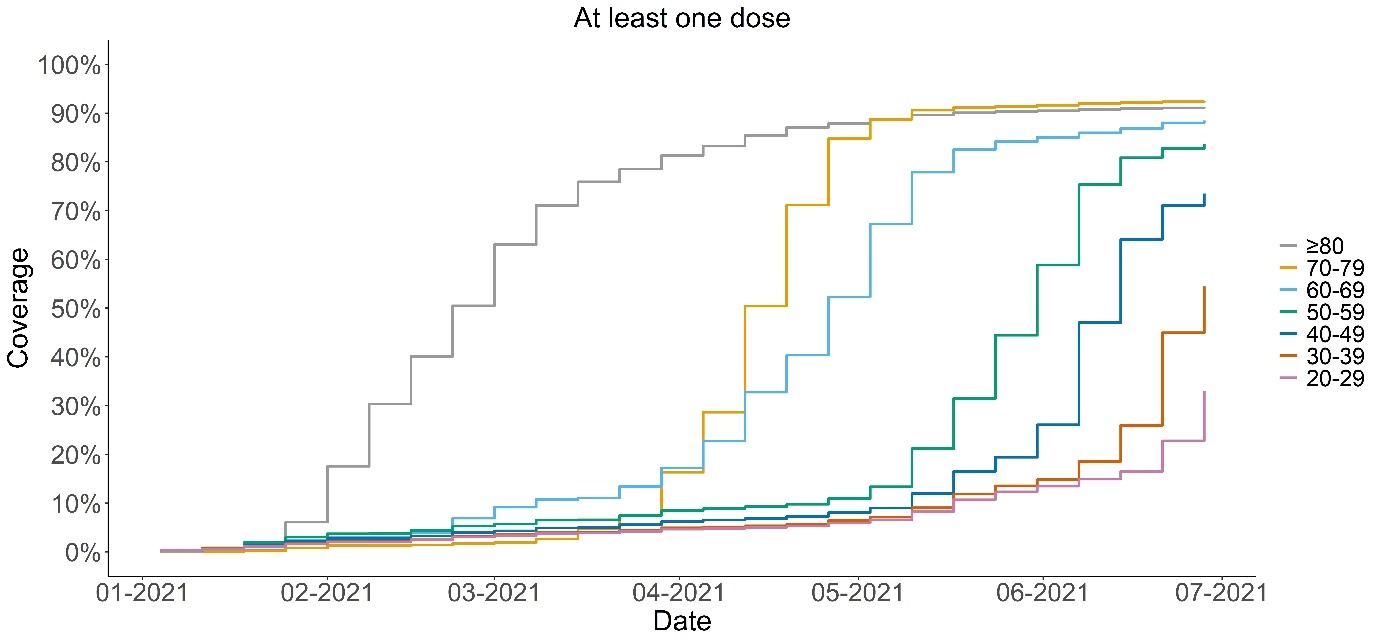
**

**B**


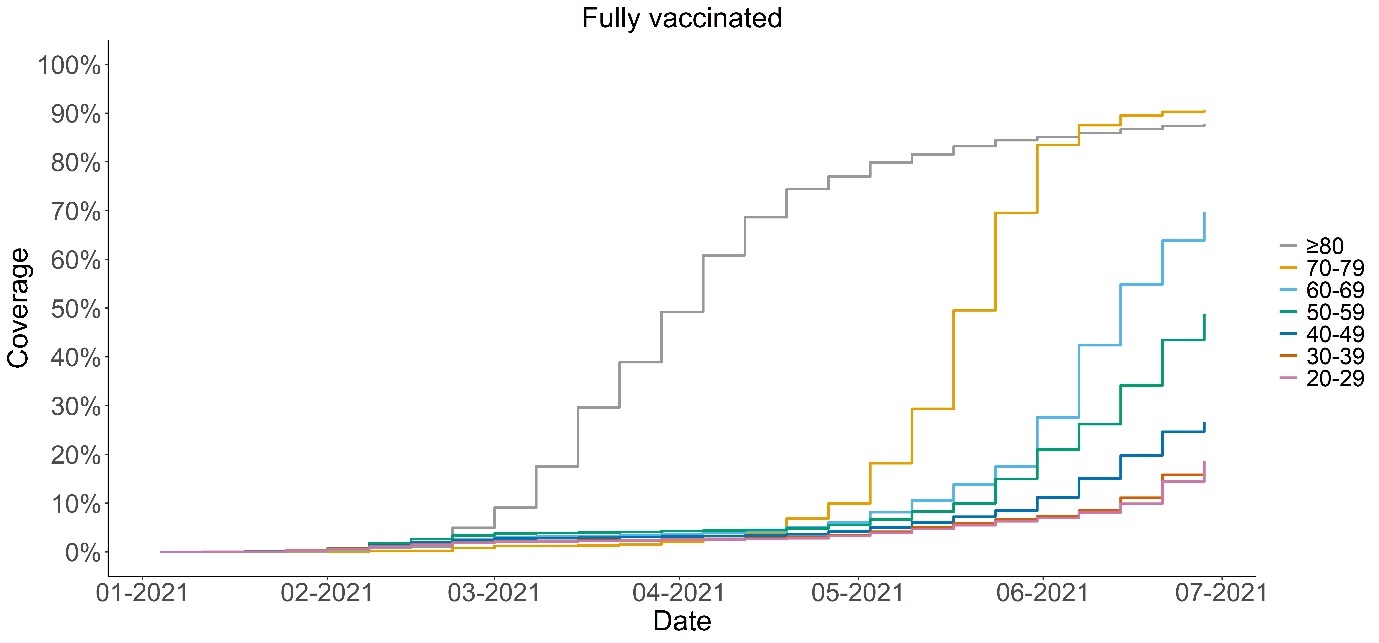


Figure S2: The vaccination coverage of the COVID-19 vaccination programme in the Netherlands over time and by age-group between January 2021 and December 2021. Panel A shows the vaccination coverage of at least one dose, and panel B for the complete vaccination series. Figures are adapted from [2].

## References

1. van Asten L, Harmsen CN, Stoeldraijer L, Klinkenberg D, Teirlinck AC, de Lange MMA, Meijer A, van de Kassteele J, van Gageldonk-Lafeber AB, van den Hof S *et al*. Excess Deaths during Influenza and Coronavirus Disease and Infection-Fatality Rate for Severe Acute Respiratory Syndrome Coronavirus 2, the Netherlands. Emerg Infect Dis 2021, 27(2):411-420.

2. National Institute for Public Health and the Environment. COVID-19 vaccination participation in the Netherlands [In Dutch] 2021 <https://www.rivm.nl/sites/default/files/2021-07/COVID-19_Vaccinatie_Schattingen_WebSite_rapport_20210705_1549_def4.pdf>. Accessed at 22 June 2023.
